# Regulation of alternative splicing in response to temperature variation in plants

**DOI:** 10.1093/jxb/erab232

**Published:** 2021-05-24

**Authors:** Sheeba John, Justyna Jadwiga Olas, Bernd Mueller-Roeber

**Affiliations:** 1 University of Potsdam, Institute of Biochemistry and Biology, Karl-Liebknecht-Straße, Haus, Potsdam, Germany; 2 Max Planck Institute of Molecular Plant Physiology, Am Muehlenberg, Potsdam, Germany; 3 Center of Plant Systems Biology and Biotechnology (CPSBB), Plovdiv, Bulgaria; 4 University Paris-Sud, France

**Keywords:** Alternative splicing, ambient temperature, cold, heat, plants, stress adaptation

## Abstract

Plants have evolved numerous molecular strategies to cope with perturbations in environmental temperature, and to adjust growth and physiology to limit the negative effects of extreme temperature. One of the strategies involves alternative splicing of primary transcripts to encode alternative protein products or transcript variants destined for degradation by nonsense-mediated decay. Here, we review how changes in environmental temperature—cold, heat, and moderate alterations in temperature—affect alternative splicing in plants, including crops. We present examples of the mode of action of various temperature-induced splice variants and discuss how these alternative splicing events enable favourable plant responses to altered temperatures. Finally, we point out unanswered questions that should be addressed to fully utilize the endogenous mechanisms in plants to adjust their growth to environmental temperature. We also indicate how this knowledge might be used to enhance crop productivity in the future.

## Teaser

Alternative splicing leads to diverse mature RNAs from a given gene. Here, we review current knowledge of alternative splicing triggered by a variation in environmental temperature in plants.

## Introduction

In natural environments, plants are repeatedly or continuously exposed to a wide range of temperatures. Temperature is a key factor affecting plant growth and development. Apart from the daily fluctuations in ambient temperature (which additionally changes throughout the lifetime of a plant), plants are often exposed to ‘unpredictable’ extreme temperatures, such as unusual cold or heat, during different times of the year. The current rise in average global temperature has a huge impact on plant growth and agricultural production, often leading to a decline in crop yield. Due to thermal stresses, every degree Celsius rise in temperature above current temperature may potentially decrease yield by 3–7% for major crops like wheat, rice, and maize (Zhao *et al.* 2017). Thus, from an agronomic point of view, it is essential to unravel how plants perceive and respond to fluctuations in temperature. As plants are sessile organisms they cannot relocate to escape a stress. Instead, to avoid or minimize the detrimental effects of stressful conditions such as high or low temperature, plants rely on mechanisms established during evolution to effectively respond and ensure survival and reproductive growth. One such mechanism is alternative splicing (AS) of precursor mRNA (pre-mRNA) in response to environmental perturbations including changes in temperature (Filichkin *et al.*, 2015a; Shang *et al.*, 2017; Laloum *et al.*, 2018). Recent studies suggest that alternative pre-mRNA splicing may serve as a ‘molecular thermometer’ in the temperature-controlled adaptation of plants, allowing them to appropriately adjust transcript abundance (Capovilla *et al.*, 2015). AS is a common phenomenon in many organisms. For example, primary transcripts of 60% of the genes in *Drosophila melanogaster* undergo AS (Graveley *et al.*, 2011), and in humans, primary transcripts of more than 95% of genes are affected by differential splicing (Pan *et al.*, 2008). In Arabidopsis, the increased utilization of next-generation sequencing technologies in recent years has shown that up to 70% of the plant multi-exon genes generate more than a single transcript by AS, and that previous technical approaches such as microarray-based analyses had underestimated the proportion of AS-affected genes (Calixto *et al.*, 2018; Li *et al.*, 2020*a*).

Although temperature regulates diverse biological processes in plants and triggers AS events, the molecular mechanisms controlling temperature-dependent AS are still poorly understood. Here, we review the current knowledge about the effects of environmental temperature (low, high, and intermediate) on AS in plants. First, we briefly describe the mechanism of splicing regulation. We then highlight examples of how AS enables favourable plant responses to altered temperatures. Finally, we point out currently unsolved questions to be addressed, and outline how this knowledge might be used to enhance crop productivity in the future.

## The mechanism of alternative splicing

**Fig. 1.**
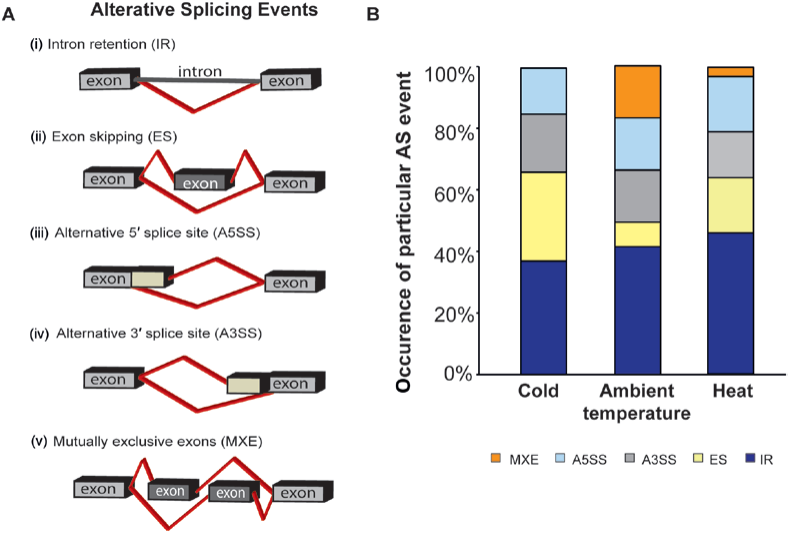
Alternative splicing (AS) events in plants. (A) Types of splicing events (see also Verhage *et al.*, (2017)). (B) Percentage of different AS events reported to date in response to cold, changes in ambient temperature, and heat in plants. Data were extracted from PubMed Central, December 2020 (https://pubmed.ncbi.nlm.nih.gov/). A3SS, alternative 3′ splice site selection; A5SS, alternative 5′ splice site selection; ES, exon skipping; IR, intron retention; MXE, mutual exclusion of exons.

Constitutive splicing of pre-mRNA is controlled by a large ribonucleoprotein complex, called the spliceosome, which leads to the removal of non-coding introns to join the flanking exons and, thereby, assembles one mature transcript (Deckert *et al.*, 2006; Moore and Proudfoot, 2009; Kornblihtt *et al.*, 2013). The spliceosome core components are supported by the serine/arginine-rich (SR) proteins and heterogeneous nuclear ribonucleoproteins that are responsible for splice site selection by binding to *cis*-regulatory elements located in exons or introns, thus activating or repressing the splicing process (Reddy, 2007; Kornblihtt *et al.*, 2013). Selection of alternative splice sites in a single type of pre-mRNA leads to AS, and thereby to the production of multiple mature mRNA isoforms (Reddy, 2007). Five basic types of AS are observed in plants depending on the selection of splice sites at the same pre-mRNA. Elimination of splice site selection generates intron retention (IR), exon skipping, or mutual exclusion of exons, while the selection of distinct splice sites result in the generation of alternative 5′ or 3′ splice sites (Fig. 1A) (Reddy *et al.*, 2013; Posé *et al.*, 2013). In this way, the same single pre-mRNA may undergo different AS events resulting in the formation of two or more mature transcripts encoding different protein isoforms, and these may functionally differ from one another (Nilsen and Graveley, 2010).

AS has the potential to change the number of protein variants (and, hence, their biochemical properties) encoded by a given genome. It can lead to the production of proteins with or without functional domains, thereby resulting in protein variants with a loss or gain of function, or to proteins with changed biochemical or cellular properties that may affect subcellular localization, stability, and function. In plants, a large number of transcripts of genes are alternatively spliced during stressful and non-stressful changes in the environment. Experimental evidence shows that those changes in response to environmental stresses can be beneficial for plants, allowing them to rapidly adjust the transcript abundance of essential genes including key regulators involved in stress response, thereby promoting stress tolerance (Fig. 1B). In plants, approximately 40% of all AS events in response to changes in temperature are due to IR, making it the most common type of AS (Fig. 1B). IR was found to be the predominant AS event in plants exposed to cold, moderate temperature changes, and heat stress, while the occurrence of other AS events varies between different temperature conditions.

**Fig. 2.**
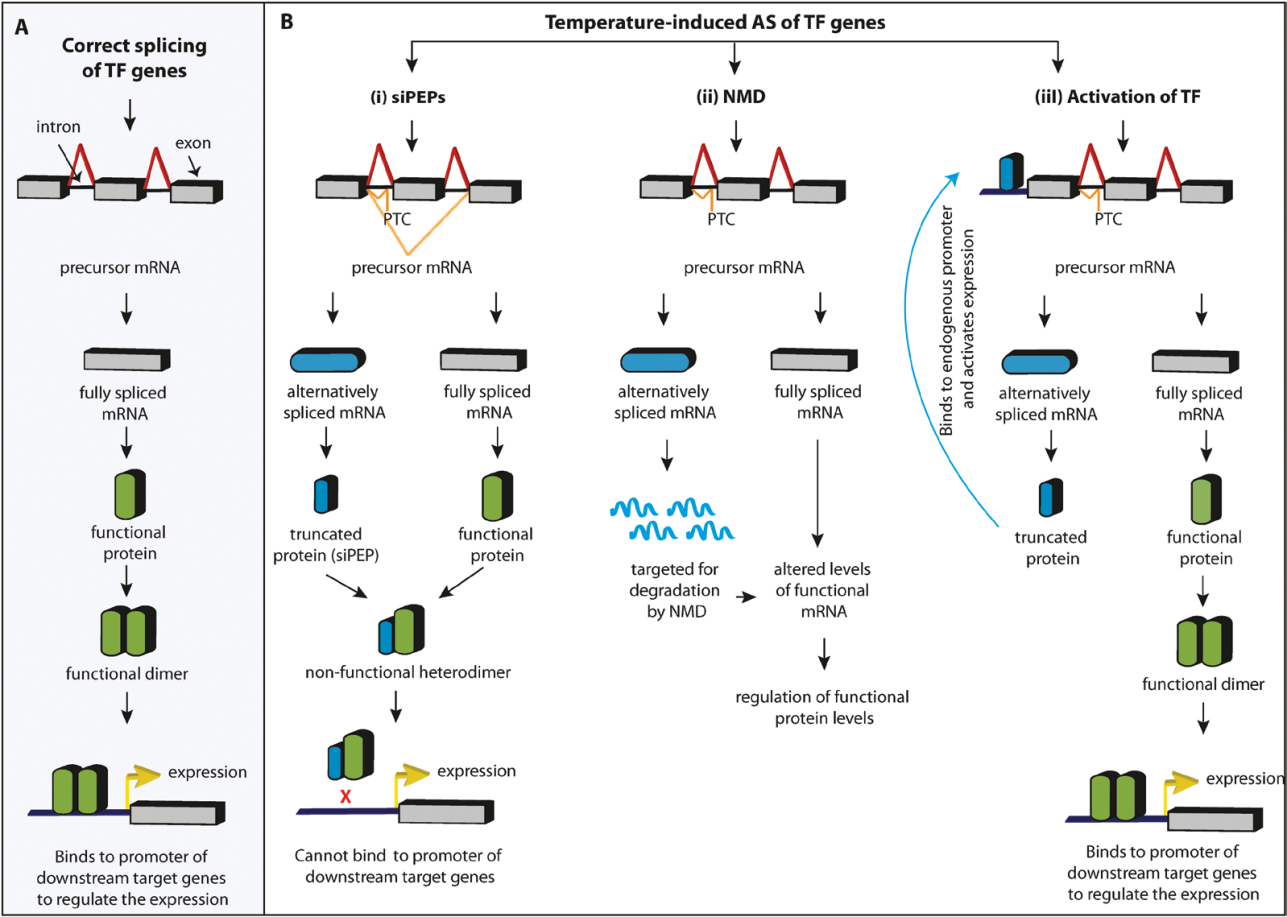
Role of temperature-induced alternative splicing (AS) and mode of action of different splice variants. (A) Regulation of down-stream target genes when the transcription factor (TF) transcript undergoes constitutive splicing to form a functional protein. A functional homodimer of the TF is formed that binds to the promoters of target genes to activate or repress their expression. (B) Temperature-induced AS can lead to three types of TF regulation. (i) Peptide interference by the formation of small interfering peptides (siPEPs). The alternatively spliced mRNA leads to a truncated protein that functions as an siPEP by forming a non-functional heterodimer with the functional protein; this inhibits the TF from binding to the promoters of target genes to affect their expression. (ii) Nonsense-mediated decay (NMD) of alternatively spliced transcripts. Many splice variants that contain premature termination codons (PTCs) are targeted for degradation by NMD, thereby altering the transcript levels available for translation to form the functional TF protein. (iii) Activation of the TF. AS leads to the formation of a truncated protein that has the ability to bind to the promoter of its own gene and modify its expression. Red lines represent constitutive splicing and orange lines represent temperature-induced AS.

Three modes of action of AS transcripts have been reported in plants in response to temperature variations (Fig. 2). The first is peptide interference by the formation of small interfering peptides (siPEPs). In this case, the alternatively spliced mRNA is translated into a truncated protein, the siPEP, which forms a non-functional heterodimer with an otherwise functional protein, which may be a transcription factor (TF) (Seo *et al.*, 2011a; Staudt and Wenkel, 2011). The siPEPs have dimerization domains but lack other functional domains, such as DNA-binding and/or transcription regulation domains (Yun *et al.*, 2008; Seo *et al.*, 2011a). siPEPs can, therefore, act as competitive inhibitors of the targeted TFs (Seo *et al.*, 2011a; Staudt and Wenkel 2011). In contrast to RNA interference (RNAi) mediated by small interfering RNAs (siRNAs), or micro-RNAs (miRNAs) that interact with mRNAs to block their translation or induce their cellular degradation, peptide interference functions at the protein level (Ramachandran and Chen 2008; Staudt and Wenkel 2011; Naqvi *et al.*, 2012). 

The second mode of action is nonsense-mediated decay (NMD) in which alternatively spliced transcripts are degraded. Many AS events, in particular IR, lead to the introduction of a premature translation termination codon (PTC) in the spliced transcript, thereby limiting the amount of transcript encoding functional protein. Splice variants containing a PTC are often targeted for degradation by NMD (Kurihara *et al.*, 2009; Rebbapragada and Lykke-Andersen 2009; Palusa and Reddy 2010). IR thus represents a molecular mechanism to down-regulate the functional output of a gene that is already actively transcribed, thereby bypassing the need for regulating its transcription, which may be a slower response. In the case of Arabidopsis it has been predicted that about 13% of the intron-containing genes are targeted by NMD (Kalyna *et al.*, 2012), while in humans up to one-third of the transcripts generated by AS are degraded by NMD (García-Moreno and Romão, 2020). 

The third mode of action is activation of a TF. In this scenario, which has been reported for heat shock transcription factor A2 (HSFA2) in Arabidopsis, AS leads to the formation of a truncated, and C-terminally modified, TF protein with an extra leucine-rich motif. The modified TF has the capacity to bind to its own promoter to activate *HSFA2* transcription (Liu *et al.*, 2013).

## Alternative splicing in response to low temperature

**Fig. 3.**
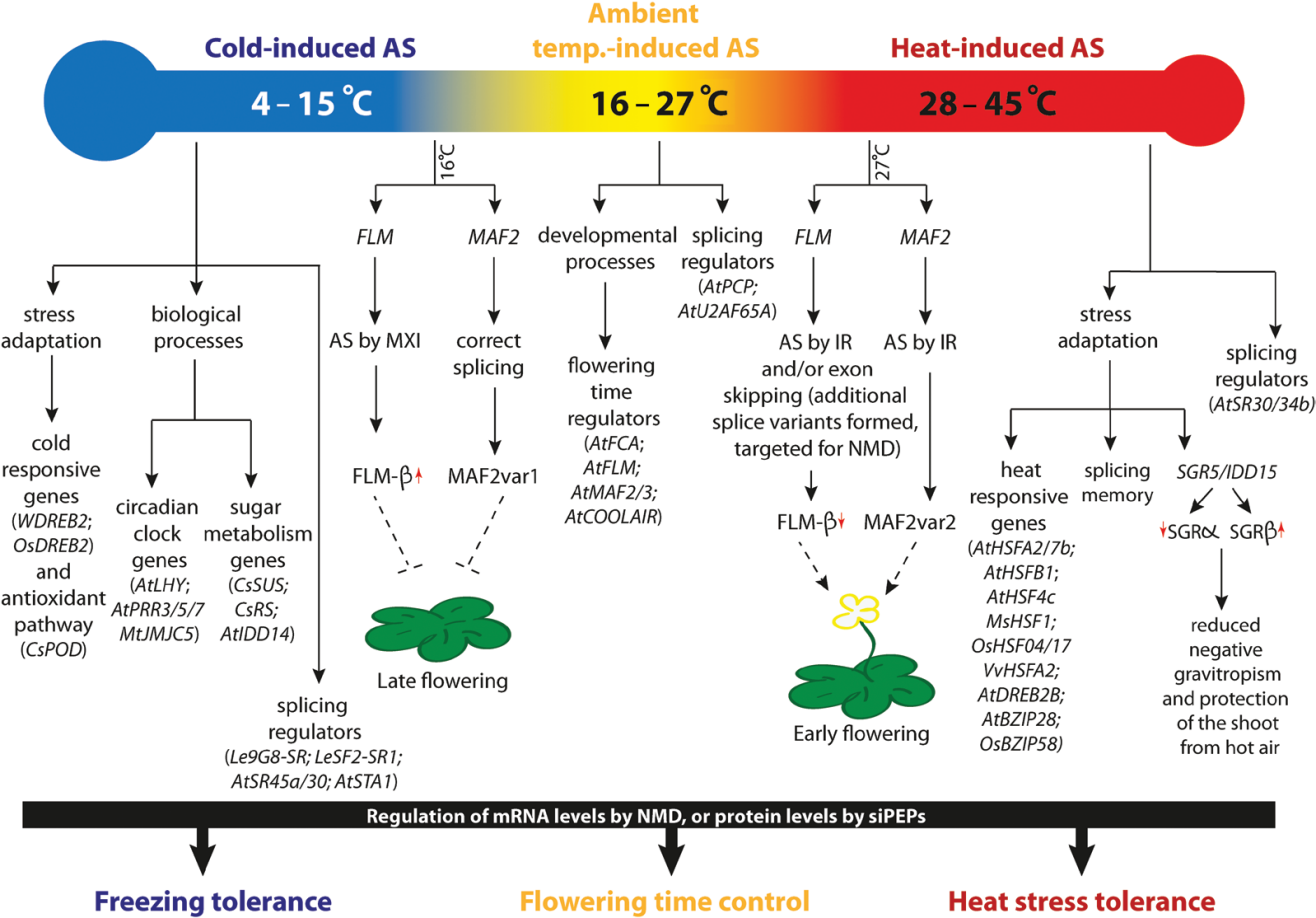
Temperature-induced alternative splicing (AS) under different temperature conditions resulting in the plant’s adaptation. Overview of the genes undergoing AS under diverse temperature regimes like cold (around 4–15 °C), changes in ambient temperature (16–27 °C), and heat stress (28–45 °C), and the resulting physiological responses. AS in response to changes in temperature (including extreme temperatures) has been shown to play an important role in improving plant performance and stress tolerance. As an example, AS of the flowering time genes *FLM* and *MAF2* in response to a change in ambient temperature regulates the transition to flowering and reproductive growth in plants. A higher ambient temperature (27 °C) induces flowering while a lower temperature (16 °C) represses flowering. Dashed lines indicate indirect responses. The red arrows indicate up-regulation (upward arrow) or down-regulation (downward arrow). IR, intron retention; MXI, mutually exclusive incorporation; NMD, non-sense mediated decay; siPEPs, small interfering peptides.

Cold is one of the major abiotic stresses affecting growth and development in higher plants, leading to reduced crop yields (Xin and Browse, 2000). Plants differ in their tolerance to chilling (0–15 °C) and freezing (<0 °C) temperatures. Many important tropical and subtropical crop plants like rice, corn, and tomato are sensitive to chilling stress whereas temperate crops like wheat, barley, and rye are better adapted to survive freezing temperatures (Chinnusamy *et al.*, 2007; Zhu *et al.*, 2007). In higher plants, low temperature (4–15 °C) has a huge impact on splicing regulation (Fig. 3) (Calixto *et al.*, 2018; Chechanovsky *et al.*, 2019; Li *et al.*, 2020*a*, *b*). AS in response to cold appears to occur very rapidly (in the range of minutes), and small shifts in the cold temperature range result in changes in the number of transcripts undergoing AS (Calixto *et al.*, 2018; Gallegos, 2018). It is estimated that approximately 33% of the cold-responsive transcripts are alternatively spliced (Calixto *et al.*, 2019). Here, we summarize recent findings that describe how low temperature induces AS in different plants.

In general, the response to cold is regulated by the CBF–COR hierarchical network with C-REPEAT/DEHYDRATION-RESPONSIVE ELEMENT BINDING FACTORS (CBF/DREBs) and their downstream targets, *COLD-REGULATED* (*COR*) genes, as the central components (Badawi *et al.*, 2007; Mao and Chen 2012). The transcripts of *DREB* genes undergo AS in response to cold whereby the changes in temperature regulate the abundance of particular isoforms. For example, the wheat *WDREB2* gene, a homologue of the Arabidopsis *DREB2A* and *DREB2B* genes, undergoes AS by exon skipping to produce three splice variants at 4 °C (Egawa *et al.*, 2006). Similarly, the rice *DREB2*-type gene *OsDREB2B* is alternatively spliced into two active variants, *OsDREB2B1* and *OsDREB2B2*, at low temperatures (Matsukura *et al.*, 2010). In addition to DREBs, the *COR* genes undergo AS in response to cold. In durum wheat (*Triticum durum*), the transcripts of two early *COR* (*e-COR*) genes putatively encoding a ribokinase and a C3H2C3 RING-finger protein are alternatively spliced to retain a subset of introns in the mature mRNA during the cold period (Mastrangelo *et al.*, 2005). In the tea plant (*Camellia sinensis*), the *CsCOR* gene was also found to undergo AS to form a truncated protein at low temperature; however, whether this shortened protein has a biological function has not been reported (Li *et al.*, 2020b).

It is interesting to note that AS affects not only transcripts of genes that regulate the cold response in plants, but also a range of genes involved in plant growth and development including the circadian clock. In Arabidopsis, the expression of isoforms of many clock components, e.g. *LATE ELONGATED HYPOCOTYL* (*LHY*), *PSEUDO-RESPONSE REGULATOR* 3 (*PRR3*), *PRR5*, *PRR7*, *PRR9*, and *TIMING OF CAB* (*TOC1*), is controlled by AS by IR (Filichkin *et al.*, 2015b). Similarly, cold-dependent IR of *CIRCADIAN CLOCK-ASSOCIATED 1* (*CCA1*) leads to the formation of the two isoforms CCA1α and CCA1β. Here, the retention of the fourth intron produces the CCA1β isoform that forms a non-functional heterodimer with CCA1α and inhibits CCA1α’s activity. In this way, CCA1β acts as a siPEP. AS of the *CCA1* transcript is suppressed by cold, which releases *CCA1α* repression and allows it to be fully functional in regulating freezing tolerance in Arabidopsis (Park *et al.*, 2012; Seo *et al.*, 2012). In *Medicago truncatula*, cold induction results in the formation of four alternatively spliced variants of *MtJMJC5*, which encodes a JmjC domain-containing protein, a circadian clock component (Shen *et al.*, 2016). Also, in a commercial sugarcane variety (*Saccharum* hybrid, SP80-3280), the temperature- and organ-dependent AS of five clock genes, *ScLHY*, *ScPRR37*, *ScPRR73*, *ScPRR95*, and *ScTOC1*, was observed (Dantas *et al.*, 2019). These findings suggest a strong correlation between temperature and AS events in circadian clock genes.

In addition to the already mentioned AS events in clock genes, transcripts of genes involved in flowering time regulation are alternatively spliced in response to prolonged cold exposure (Guan *et al.*, 2013; Wang *et al.*, 2019). The main mechanism of vernalization-mediated flowering requires repression of the transcription of *FLOWERING LOCUS C* (*FLC*), a MADS-box TF that acts as a negative regulator of flowering (Sheldon *et al.*, 2000). The cold-dependent silencing of *FLC* is controlled by the long non-coding anti-sense RNA *COLD INDUCED LONG ANTISENSE INTRAGENIC RNA* (*COOLAIR*) produced from the *FLC* locus (Swiezewski *et al.*, 2009; Sun *et al.*, 2013). The temperature-dependent AS of *COOLAIR* subsequently regulates the expression of *FLC* through co-transcriptional coupling mechanisms (Swiezewski *et al.*, 2009; Hornyik *et al.*, 2010; Marquardt *et al.*, 2014), providing plants with the ability to measure temperature and to integrate this external information to regulate flowering time.

Furthermore, links between AS, the response to cold stress, and plant metabolism were reported. In particular, in *C. sinensis*, a large number of AS events occurs in transcripts associated with sugar metabolism and antioxidant pathways, such as *SUCROSE SYNTHASE* (*CsSUS*), *RAFFINOSE SYNTHASE* (*CsRS*), and *PEROXIDASE* (*CsPOD*) (Li *et al.*, 2020b). These pathways contribute to establishing cold tolerance by protecting membrane structures, reducing oxidative damage through the induction of reactive oxygen species (ROS)-degrading enzymes, and the accumulation of sugars that can have a protective function at high concentration (Uemura and Steponkus, 2003; Sun *et al.*, 2010; Rai *et al.*, 2013). Similarly, in Arabidopsis, the primary transcript of *INDETERMINATE DOMAIN 14* (*IDD14*), a regulator of starch metabolism, undergoes AS by IR to form the functional IDD14α isoform and the truncated form IDD14β (Seo *et al.*, 2011b). IDD14α and IDD14β form a heterodimer that displays reduced binding capacity to the promoter of the *QUA-QUINE STARCH* (*QQS*) gene that regulates starch accumulation. Overexpression of *IDD14α* leads to retarded growth, similar to *QQS* overexpression. As *IDD14β* overexpression rescues the *IDD14α* overexpression phenotype, AS of the *IDD14* transcript may be a cold adaptation strategy for the plant to modulate starch accumulation to withstand the cold conditions (Seo *et al.*, 2011b). These findings suggest a possible link between carbohydrate metabolism and low-temperature signalling mediated by AS. Also in potato (*Solanum tuberosum*), sugar metabolism genes undergo AS. Here, cold-induced AS by exon skipping occurs in the transcripts of one of the plant’s *INVERTASE* genes responsible for the conversion of sucrose to glucose and fructose (Bournay *et al.*, 1996). The accumulation of these reducing sugars in tubers stored under cold conditions leads to reduced tuber quality in many cultivars. This phenomenon is known as low-temperature sweetening (Davies *et al.*, 1989). Here, invertase inhibitors bind to invertases to inactivate them (Hothorn *et al.*, 2010). Interestingly, one of the most abundant forms of invertase inhibitor genes expressed in potato tubers, *INH2*, is regulated by AS. During cold storage of potato tubers of cultivars resistant to cold-induced sweetening, the levels of the unspliced *INH2α* transcript, which encodes the full-length INH2 protein, and hybrid *INH2β* mRNAs generated through splicing of *INH2α* and *INH1* transcripts, were higher than in tubers of sensitive cultivars. The increase in invertase inhibitor levels in the resistant cultivars may be responsible for the suppression of acid invertase activity and sucrose breakdown, thereby leading to reduced cold-induced sweetening (Brummell *et al.*, 2011). Also, sugar transporter genes, e.g. *SUGARS WILL EVENTUALLY BE EXPORTED TRANSPORTER 17* (*SWEET17*) in *C. sinensis* undergo AS in response to cold (Yao *et al.*, 2020). Interestingly, a metabolic or physiological reprogramming of the transcriptome as a result of AS was also observed in animals such as *Caenorhabditis elegans*, mouse, and human, where genes involved in pyruvate metabolism, glycolysis, and gluconeogenesis were found to be alternatively spliced (Tabrez *et al.*, 2017; Biamonti *et al.*, 2018).

A recent study showed that 46 of the 379 long non-coding RNAs (lncRNAs) in Arabidopsis are alternatively spliced in response to cold (of which half are also differentially expressed after cold treatment), suggesting an important regulatory mechanism involved in cold acclimation and freezing tolerance (Calixto *et al.*, 2019). For example, the transcript of *TAS1a* is alternatively spliced upon exposure to cold, without a major change in gene expression level. The non-spliced *TAS1a* transcript, which is more abundant at normal growth temperature, contains a *miR173* binding site in its intron; binding of *miR173* to the non-spliced intron sequence causes the formation of transacting siRNAs (tasiRNAs). The splicing of the primary *TAS1a* transcript at low temperature removes its intron, thereby eliminating the *miR173* binding site and leading to a reduction of the level of siRNAs. The data demonstrate that AS regulates the production of siRNAs in response to cold stress (Calixto *et al.*, 2019). In addition, various studies provide evidence that cold induces AS in a tissue-specific manner. For example, tissue-specific cold-induced AS occurs in the wild rice *Oryza longistaminata*, which is tolerant to cold non-freezing temperatures, unlike *Oryza sativa*, which is chilling stress-sensitive. Transcriptome profiling of shoots and rhizomes of *O. longistaminata* subjected to chilling stress revealed that cold-induced AS was transcript-specific in these tissues. Most of the chilling-induced genes undergoing AS only in shoots are involved in photosynthesis and the regulation of gene expression, whereas those undergoing AS only in rhizomes are mainly involved in stress signal transduction. This observation suggests that tissue-specific AS may play an important role in regulating cold acclimation in *O. longistaminata* (Zhang *et al.*, 2017).

In summary, compelling evidence from various studies demonstrates that a large number of AS events observed in plants in response to cold temperature are not random. In fact, those events contribute to the acquisition of cold acclimation in plants, allowing them to develop cold tolerance.

## Alternative splicing induced by moderate changes in ambient temperature

In addition to low temperature, moderate changes in ambient temperature may trigger AS (Fig. 3) (Verhage *et al.*, 2017). For example, transcripts of Arabidopsis genes encoding the RNA-binding protein PUMILIO 23 (PUM23) and three other regulatory proteins, i.e. mitochondrial EMBRYO DEFECTIVE 3114 (EMB3114), SNF1 KINASE HOMOLOG 11 (AKIN11), and FYD (Streitner *et al.*, 2013), are subject to AS at small changes in ambient temperature (from 16 °C to 20 °C, from 20 °C to 24 °C, and from 20 °C to 16 °C) (Streitner *et al.*, 2013).

AS is also involved in regulating flowering time in response to fluctuating ambient temperature. The temperature-dependent AS observed in the transcript of *FLOWERING CONTROL LOCUS* A (*FCA*), which encodes an RNA-binding protein involved in the biosynthesis of the flowering time regulator *microRNA172* (*miR172*) (Macknight *et al.*, 1997; Jung *et al.*, 2012), is a well-known example. The *FCA* mRNA undergoes AS so that functional FCA protein is more abundant at 23 °C than 16 °C, which leads to an accumulation of *miR172* at the higher temperature (Jung *et al.*, 2012).

Other flowering time regulators are also controlled by ambient temperature-triggered AS, including FLOWERING LOCUS M (FLM), a MADS-domain TF (Scortecci *et al.*, 2001; Balasubramanian *et al.*, 2006). FLM modulates flowering time by creating a repressor complex with FLC or SHORT VEGETATIVE PHASE (SVP) proteins in a wide range of temperatures. Interestingly, the *FLM* transcript undergoes alternative splicing in response to changes in ambient temperature (Balasubramanian *et al.*, 2006). *FLM-β* and *FLM-δ* are the predominant splice variants formed by the mutual exclusion of exons: while exon 2 is maintained in the *FLM-β* transcript, exon 3 is retained in the *FLM-δ* transcript (Lee *et al.*, 2013; Posé *et al.*, 2013). Low ambient temperature (16 °C) favours the expression of the repressive isoform *FLM-β*, and its expression decreases when ambient temperature increases (27 °C) (Lee *et al.*, 2013; Posé *et al.*, 2013). FLM-β protein forms a complex with SVP, a MADS-domain TF that actively represses flowering by binding to, for example, the promoter of the flowering time integrator *SUPPRESSOR OF OVEREXPRESSION OF CONSTANS 1* (*SOC1*) (Posé *et al.*, 2013). Although initial research had indicated an important role of the FLM-δ isoform for flowering control, as a competitor of FLM-β in its interaction with SVP, follow-up research revealed a less important role of FLM-δ (Lutz *et al.*, 2015; Sureshkumar *et al.*, 2016; Lutz *et al.*, 2017). Apart from *FLM-β* and *FLM-δ*, additional *FLM* splice variants are generated in Arabidopsis Col-0, particularly at higher ambient temperatures (Sureshkumar *et al.*, 2016; Capovilla *et al.*, 2017). Most of the additional splice variants harbour PTCs and are targeted for degradation by NMD, which in consequence leads to a decrease in the level of transcripts available for translation into the functional isoform FLM-β at higher ambient temperatures. Furthermore, the SVP protein is degraded at higher ambient temperatures (Lee *et al.*, 2013). This decrease in the FLM–SVP repressor complex promotes flowering at elevated temperatures (Sureshkumar *et al.*, 2016). To further elucidate the role of FLM-β and FLM-δ in flowering, CRISPR-Cas9-generated mutants lacking the second (*FLM-∆E2*, *FLM-δ*) or third (*FLM-∆E3*, *FLM-β*) exon of the *FLM* transcript were studied (Capovilla *et al.*, 2017). A comparison of the genome-edited plants with the *flm* loss-of function mutant showed that expression of *FLM-δ* alone did not promote flowering while expression of *FLM-β* delayed flowering. Taken together, the results suggest that FLM-δ is unlikely to promote flowering, while temperature-induced AS regulates the levels of FLM-β, which plays a crucial role in flowering time regulation (Fig. 3).

The balance between the FLM-β and FLM-δ isoforms is regulated by splicing factors like the U2 AUXILIARY FACTORs ATU2AF65A and ATU2AF65B, the GLYCINE-RICH PROTEINs ATGRP7 and ATGRP8, and SPLICING FACTOR 1 (ATSF1) (Lee *et al.*, 2017; Park *et al.*, 2019; Steffen *et al.*, 2019). Recently, the CYCLIN-DEPENDENT KINASE G2 (CDKG2)–CYCLIN L1 (CYCL1) complex has also been shown to regulate this balance and to be required for the correct processing of the alternative introns 1 and 4 in *FLM* pre-mRNA (Nibau *et al*., 2020). When the CDKG2–CYCL1 complex was absent, expression of the *FLM-δ* isoform increased while expression of the *FLM-β* variant decreased in a temperature-dependent manner, resulting in an early-flowering phenotype (Nibau *et al*., 2020). Interestingly, the CDKG2–CYCL1 complex also regulates the temperature-dependent splicing of another CDK, i.e. CDKG1, which regulates AS of the splicing factor *ATU2AF65A* transcript (Cavallari *et al.*, 2018). *ATU2AF65A* undergoes AS when ambient temperature is lowered from 23 °C to 16 °C (Verhage *et al.*, 2017). This observation indicates that apart from their primary function in the progression of the cell cycle, CDKs may play an essential role in linking temperature sensing mechanisms with the AS of specific transcripts.

Another flowering time component subject to AS is *MADS AFFECTING FLOWERING 2* (*MAF2*), a gene closely related to *FLM* at the sequence level. Different AS events occur in the *MAF2* primary transcript. However, only one of them leads to the formation of a functional, full-length MIKC-type MADS-domain TF that represses flowering (Rosloski *et al.*, 2013). The expression of this splice variant is elevated at low ambient temperature (16 °C), and the protein it encodes interacts with SVP to repress flowering. Interestingly, at higher ambient temperatures (21–27 °C), the *MAF2* primary transcript undergoes AS by IR to introduce a PTC (Rosloski *et al.*, 2013). This isoform encodes a protein incapable of interacting with SVP and, consequently, to inhibit flowering. A progressive temperature-dependent early-flowering phenotype has been observed in Arabidopsis plants, which flower earlier at 27 °C than at 21 °C, and earlier at 21 °C than at 16 °C (Airoldi *et al.*, 2015). At higher ambient temperatures, AS triggers the increased production of *MAF2* and *FLM* isoforms, which lack the repressive function that may be responsible for the decrease in flowering repression. Similarly, also *MAF3* undergoes AS in response to changes in ambient temperature (Verhage *et al.*, 2017). Among the several AS products derived from the primary *MAF3* transcript, only the transcript that includes a skipping of exon 2 shows temperature sensitivity (Verhage *et al.*, 2017).

There is increasing evidence that AS in response to changes in ambient temperature is not only limited to genes involved in flowering time regulation, but also affects other developmental genes. For example, the splice regulator PORCUPINE (PCP) has recently been reported to be a temperature-specific regulator of development in Arabidopsis (Capovilla *et al.*, 2018). When *pcp-1* knockout plants grown at permissive ambient temperature (23 °C) were shifted to a lower temperature (16 °C), growth was arrested and plants were rendered male sterile. When the permissive temperature was re-established, the *pcp-1* loss-of-function mutant restored the wild type-like phenotype. At low ambient temperature (16 °C), *pcp-1* plants showed aberrant shoot apical meristems, and altered lateral organ formation. In a proteome study, two variants of the PCP protein, PCP-α and PCP-β, which are encoded by alternatively spliced *PCP* transcripts, were identified (Ito *et al.*, 2011; Capovilla *et al.*, 2018). The constitutive overexpression of *PCP-α*, but not *PCP-β*, rescues the *pcp-1* phenotype at 16 °C. The two splice variants differ from each other only in the extreme 3′ part of the protein-coding region, indicating that the C-terminal region of the PCP protein may be imperative for its function (Capovilla *et al.*, 2018).

It is important to note that among the genes in Arabidopsis undergoing AS due to elevated ambient temperature, approximately 96% harbour a histone H3 lysine 36 tri-methylation (H3K36me3)-enriched region in their gene body. Several flowering time regulators (such as *FLM*, *MAF2*, and *FCA*) and circadian clock components (*PRR3* and *PRR7*) affected by temperature-induced AS harbour this mark (Pajoro *et al.*, 2017). In addition, lack of histone methyltransferases involved in the deposition of H3K36me3 marks led to altered AS upon a temperature shift from 16 °C to 25 °C. Mutants affected in writing, erasing, and reading the H3K36me3 mark showed altered elevated temperature-induced flowering. Therefore, the H3K36me3 mark plays an important role in regulating ambient temperature-induced AS of biological relevance. The results also demonstrate that AS and epigenetic regulation of chromatin are coupled (Pajoro *et al.*, 2017). Evidence that AS affects the epigenetic landscapes of cells, thereby modulating gene expression, has also been obtained in mammals. For example, LYSINE-SPECIFIC DEMETHYLASE 1 has been reported to repress and activate gene expression programmes in neurons by mediating histone H3K4me1/2 and H3K9me1/2 demethylation, respectively (Laurent *et al.*, 2015).

## Heat-regulated alternative splicing

Heat stress is another abiotic stress that adversely affects plant growth and reduces crop yields. Each crop has a different threshold temperature (above which growth and development are severely affected). For example, a temperature threshold of 35 °C has been reported for rice, and 33–38 °C for maize. The susceptibility to high temperature can also depend on the stage of development of the plant. For example, the temperature threshold in wheat was observed to be 20–30 °C for vegetative growth and 15 °C for reproductive growth, whereas in tomato the temperature threshold was observed to be 37 °C for vegetative growth and 28–30 °C for reproductive growth (Janni *et al.*, 2020). Given the current level of knowledge of AS in plants in response to high temperature, it is predicted that ~46, 46, and 55% of intron-containing genes in Arabidopsis undergo AS in response to mild, severe, or extreme/lethal changes in temperature, respectively (Ling *et al.*, 2018). Here, we summarize recent findings that describe how heat stress induces AS in different plant species that in turn can lead to plant adaptation to higher temperatures.

The heat stress response is controlled by members of the heat shock factor (HSF) transcription factor family that activate (or repress) *HEAT SHOCK PROTEIN* (*HSP*) genes. The HSPs in turn function as molecular chaperones to protect the proteome against the negative effects of heat stress (Zhang *et al.*, 2010; Wu *et al.*, 2013). Interestingly, HSFs are strongly impacted by splicing regulation in the response to heat stress in plants. In the *Drosophila HSF* (*dHSF*), both heat- and cold-induced AS are observed, in which the abundance of the *dHSFb* isoform increases upon heat exposure, while that of the *dHSFd* isoform increases upon cold exposure, which may lead to the induction of different HSPs (Fujikake *et al.*, 2005). AS of *HSFs* in response to cold has not yet been reported in plants. In Arabidopsis, various *HSFs*, e.g. *HSFA2*, *HSFA7b*, *HSFB1*, *HSFB2a*, and *HSF4c*, undergo extensive AS in response to heat stress by producing intron-retained splice variants (He *et al.*, 2008; Sugio *et al.*, 2009; Amano *et al.*, 2011; Liu *et al.*, 2013; Cheng *et al.*, 2015; Jiang *et al.*, 2017; Zhang *et al.*, 2020). For example, at 22 °C the *HSFA2* transcript is fully spliced, while at a moderately high temperature (37 °C) a 31-nucleotide-long cryptic mini-exon is derived from within the *HSFA2* intron. This leads to the generation of the splice variant *HSFA2-II* that is further degraded by NMD due to a PTC present within the mini-exon (Sugio *et al.*, 2009). Interestingly, during extreme heat (45 °C) a third splice variant, *HSFA2-III*, is generated by retaining an approximately 80-nucleotide-long 5′ region of the intron in the processed mRNA. *HSFA2-III* is translated into a truncated, C-terminally modified protein, S-HSFA2. The S-HSFA2 variant binds to the *HSFA2* promoter, thereby creating a positive auto-regulatory loop controlling *HSFA2* expression (Liu *et al.*, 2013). Of note, alternatively spliced *HSF* transcripts are observed in different plant species, e.g. *Medicago sativa HSF1*, *Potamogeton malaianus* and *P. perfoliatus HSFA2a2*, rice (*Oryza sativa*) *HSFA2d*, maize (*Zea mays*) *ZmHSF04* and *ZmHSF17*, and grape (*Vitis vinifera*) *HSFA2* (He *et al.*, 2008; Sugio *et al.*, 2009; Amano *et al.*, 2011; Liu *et al.*, 2013; Cheng *et al.*, 2015; Jiang *et al.*, 2017; Zhang *et al.*, 2020). In lily (*Lilium* spp.), *LlHSFA3B* undergoes heat-induced AS to generate splice variant *LlHSFA3B-III* (Wu *et al.*, 2019). The LlHSFA3B-III protein appeared to be transcriptionally inactive in a yeast test system. A green fluorescent protein (GFP)–LlHSFA3B-III fusion protein accumulated in both the nucleus and cytoplasm. Notably, LlHSFA3B-III negatively affects the interaction of the full-length HSFA3 proteins LlHSFA3A-I and LlHSFA3B-I (Wu *et al.*, 2019). Enhanced tolerance to both salinity stress and prolonged heat treatment at 40 °C was observed in transgenic Arabidopsis and *Nicotiana benthamiana* plants expressing *LlHSFA3B-III*, while tolerance to acute heat shock at 45 °C was reduced. Furthermore, the interaction between LlHSFA3B-III with LlHSFA3A-I was found to be essential for reducing the transactivation function of LlHSFA3A-I, thereby limiting various adverse effects of increased LlHSFA3A-I accumulation such as sensitivity to salinity and heat during prolonged heat stress (Wu *et al.*, 2019). More importantly, the AS pattern for *HSF*s appears to be largely conserved between plants indicating that heat-induced AS regulation is an evolutionarily conserved phenomenon (Chang *et al.*, 2014).

It is important to note that heat stress-induced AS not only occurs in transcripts of *HSF* genes, but also in transcripts of *HSPs* and other heat stress-inducible genes such as *DREB2B* and *BZIP28* in Arabidopsis (Liu *et al.*, 2013). As in the case of *HSFs*, retention of the first intron was observed in *HSP* transcripts, suggesting a conserved mechanism of AS in response to high temperature (Neves-da-Rocha *et al.*, 2019). Interestingly, recent research suggested that STABILIZED1 (STA1), a U5-snRNP-interacting protein, acts as a high temperature-specific splicing factor involved in AS of *HSP* and *HSF* primary transcripts, i.e. *HSFA3* (Kim *et al.*, 2018). Pre-mRNA of *HSFA3*, whose transcription is induced by the upstream transcription factor DREB2A, is spliced with a contribution from STA1. The protein produced from its mature mRNA induces the expression of *HSP* genes. The findings also suggest that heat-inducible STA1 is required for the establishment of acquired, but not basal, heat stress tolerance (Kim *et al.*, 2017, 2018).

In natural environments and in agricultural fields, plants are often subject to recurrent stress. One of the strategies to cope with recurring stress is a process called ‘priming’ through which moderately stressed plants ‘prepare’ themselves to successfully withstand a later, and often more severe, stress. The period between both stresses is called the ‘memory phase’. During this phase the specific molecular-biochemical changes induced by a priming stress are maintained to allow the plant to withstand the second stress, which otherwise would be more harmful or even deadly (Hilker *et al.*, 2016). Some of the molecular mechanisms underlying molecular memory have been discovered in recent years, although a coherent picture is not yet available (Sedaghatmehr *et al.*, 2016; Luo *et al.*, 2020; Mayer and Charron, 2020).

Heat priming-induced splicing memory has been studied in Arabidopsis, where plants subjected to a priming heat stress retain the memory of constitutive splicing of transcripts of many heat-responsive genes (including *HSFs* and *HSPs*). In response to heat priming, the transcripts of *HSP* genes like *HSP21*, *HSP101*, *HSP70.10*, *HSP70.6*, *HSP90.5*, and *HSP100.3* are alternatively spliced, mostly by IR (Ling *et al.*, 2018). In primed plants, these gene transcripts undergo constitutive splicing when subjected to a second heat stress (called triggering). Unlike in primed plants, a significant splicing repression was observed in non-primed plants directly subjected to the triggering heat stress, which subsequently led to an increase in alternatively spliced isoforms of several *HSF* and *HSP* transcripts in these plants. This ‘splicing memory’ enables the primed plants to combat subsequent and otherwise lethal heat stress conditions (Ling *et al.*, 2018). Heat-induced AS may, therefore, play an important role in the thermomemory response in plants. However, the molecular mechanisms underpinning the splicing memory are unknown at present.

Interestingly, transcripts undergoing AS are not only directly involved in the heat stress response, but are also involved in plant growth and metabolism. For example, the zinc finger (ZF)-containing transcription factor SHOOT GRAVITROPISM 5 (SGR5; also called INDETERMINATE DOMAIN 15, IDD15), which is responsible for mediating initial events of gravitropic responses in inflorescence stems in Arabidopsis (Morita *et al.*, 2006), undergoes AS that is accelerated by higher temperatures. IR in the *SGR5* transcript produces two protein isoforms, the completely spliced SGR5α transcription factor and the truncated SGR5β form lacking the functional ZF motif. SGR5β binds to SGR5α to form a non-functional heterodimer that lacks DNA-binding activity and thus functions as an siPEP. Since this AS is enhanced at higher temperature, SGR5β protein accumulates during heat stress; this heat-induced accumulation of SGR5β enables shoots to curve away from the source of heat, thereby serving as an adaptive response developed to protect shoots from hot air when plants are growing in heat stress conditions (Kim *et al.*, 2016).

A second example is the rice transcription factor OsbZIP58, a regulator of various starch synthesis and storage protein genes (Hakata *et al.*, 2012; Xu *et al.*, 2020). The loss-of-function phenotype of *OsbZIP58* is characterized by reduced seed storage material, and floury and shrunken endosperms under high-temperature conditions. The *OsbZIP58* primary transcript undergoes AS by IR during heat stress to generate PTCs, leading to the increased accumulation of a truncated protein, OsbZIP58β. The newly formed OsbZIP58β protein is transcriptionally less active than OsbZIP58α derived from the fully spliced transcript. The heat-induced AS of *OsbZIP58* is more pronounced in heat sensitive rice varieties like *japonica* than in heat resistant varieties like *indica*. The production of the transcriptionally less active protein OsbZIP58β due to increased AS in *japonica* may lead to an impaired accumulation of storage materials, thereby leading to the higher heat sensitivity. The reduced AS of *OsbZIP58* in less heat sensitive *indica* may be responsible for the heat tolerance observed during grain filling in these varieties (Xu *et al.*, 2020).

In addition, heat stress-induced AS provides a mechanism for regulating miRNA processing in plants. An example for this has been reported in Arabidopsis, where intronic *miR400* is co-transcribed together with its host gene (*AT1G32583*). Under conditions of elevated temperature (37 °C, 30 min–12 h), *miR400* and the host gene transcript display opposite responses. While the abundance of the host gene transcript slightly decreased, the abundance of *miR400* primary transcript strongly increased by 12 h compared with controls; the level of mature *miR400* continuously declined. It turned out that the accumulation of *miR400* was triggered by an AS in intron 1 under higher temperatures that produced a version of the transcript from which the *miR400*-containing intron could not be spliced out. This then blocked the efficient further processing to mature *miR400* (Yan *et al.*, 2012). Overexpression of *miR400* reduces the tolerance to heat stress, although the precise molecular mode of action for this is not well known yet. However, *miR400* guides *PPR1* and *PPR2* mRNAs, which encode pentatricopeptide repeat proteins, for cleavage, which may suggest an involvement of the two proteins in the heat stress response (Yan *et al.*, 2012; Park *et al.*, 2014).

In summary, AS in response to high temperature occurs in many genes involved in the heat stress response like *HSFs*, *bZIPs*, *HSPs*, and others. The heat-induced AS of these genes leads to favourable adaptive responses in plants as seen in the case of AS in *SGR5*, *OsbZIP58*, and the heat priming-induced splicing memory.

## Temperature-induced alternative splicing of splicing regulators

In addition to the above mentioned examples, also members of the spliceosomal complex themselves undergo AS in different temperature regimes. For example, transcriptome analysis of ambient temperature-induced AS in Arabidopsis and *Brassica oleracea* ssp. *botrytis* revealed differential splicing in several classes of splicing-related genes, indicating that the whole spliceosome is affected by fluctuations in ambient temperature (Verhage *et al.*, 2017). These findings suggest a two-step model controlling ambient temperature perception through AS: first, the splicing regulator genes undergo changes in their splicing patterns, and second, the altered splicing machinery subsequently affects the splicing of downstream genes involved in the adaptation to altered temperature (Verhage *et al.*, 2017).

A good example of splicing-related genes affected by AS in response to a wide range of temperatures are those for SR proteins. In fact, transcripts of SR proteins undergo AS to generate approximately 100 distinct splice variants from 15 *SR* genes in Arabidopsis (Palusa *et al.*, 2007). The AS of some *SR* genes was found to be controlled during development and with tissue specificity. The AS of the transcripts of many *SR* genes drastically changes in response to extreme temperatures (cold and heat). The transcripts of *SR30*, *RS31a*, *RS41*, *RSZ33*, and *SCL30a* undergo heat-induced AS, while the transcripts of *SR33*/*SCL33*, *SR1*/*SR34*, and *RS40* undergo AS under both heat and cold conditions (Palusa *et al.*, 2007). Heat or cold treatments also had an impact on the recruitment of splice variants of many *SR* genes to polysomes for translation (Palusa and Reddy, 2015). Here, the functional isoforms of *SR30*, *SR34*, and *SR34a* were found to be more abundant in polysome-associated RNA of heat- and cold-treated Arabidopsis seedlings. On the contrary, many isoforms associated with polysomal RNA in the control were not recruited to polysomes under heat or cold stress conditions. One splice variant of *SR34* is recruited to polysomes only during heat stress. The differential AS in response to cold produces new splice variants of *SCL33* that are not recruited to polysomes. These splice variants may be involved in regulating the amount of functional *SCL33* mRNA. In heat-treated seedlings, the functional isoform of *SCL33* was found to be more enriched (Palusa and Reddy, 2015). In Arabidopsis, cold stress mediates the repression of the active isoforms of *SR45a* and *SR30* (Tanabe *et al.*, 2007), whereas it leads to an elevated expression level of *STABILIZED1* (*STA1*), which encodes a protein responsible for the correct splicing of the *COLD REGULATED 15a* (*COR15a*) gene transcript (Lee *et al.*, 2006). In tomato (*Solanum lycopersicum*), the splicing factor genes *Le9G8-SR* and *LeSF2-SR1* undergo AS at low temperature (Fung *et al.*, 2006).

The AS of splicing regulators that leads to the formation of different isoforms and differential protein levels in response to varying temperatures may be responsible for rapid and dynamic transcriptome changes, providing plants with development plasticity.

## Open questions regarding temperature-controlled alternative splicing

Rapid progress in exploring the regulatory aspects of AS in plants has been achieved recently. Yet, most studies have concentrated on the splicing events themselves and their impact on protein levels, while the impact of AS on the plant’s physiology has often been neglected. However, understanding the functional consequences of AS events for plants has an important practical relevance, in particular with respect to improving the resilience of crops towards climate-related environmental changes in the coming decades. In mammals the biological importance of AS is relatively well understood. A large number of human diseases are caused by mutations in splicing factors or other components of the spliceosome (Scotti and Swanson, 2016). In fact, the Human Genome Mutation Database predicts that more than one-third of the diseases caused by single nucleotide polymorphisms are due to mutations within splice sites or splicing elements (Krawczak *et al.*, 2007). Interestingly, in mammals, physiological body temperature differences of only 1 °C occurring during day–night cycles lead to functionally relevant AS events that affect the levels of TATA-box binding proteins (Tbp) and, as a consequence, global gene expression (Preußner *et al.*, 2017). Also in ectotherm insects such as *D. melanogaster*, temperature changes dramatically affect splicing patterns. Anduaga *et al.* (2019) demonstrated the presence of two thermosensitive transcript isoforms of the circadian clock gene *TIMELESS* (*TIM*). Temperature-dependent switching of the isoforms, e.g. *TIM-COLD* (18 °C) and *TIM-MEDIUM* (29 °C), mediates temperature adaptation of flies by acting as a thermometer within the circadian clock (Anduaga *et al.*, 2019).

Temperature-induced AS also plays a functional role in conferring an adaptive benefit in single-cell eukaryotic species. For example, in *Saccharomyces cerevisiae*, the *HSP70* nucleotide exchange factor *FACTOR EXCHANGE FOR SSA1 PROTEIN 1* (*FES1*) undergoes AS to produce two isoforms, *FES1L* and *FES1S*. At elevated temperature (37 °C), the expression of *FES1S* is highly induced. The protein encoded by stress-inducible *FES1S* localizes to the cytosplasm and plays an important role in proteasomal degradation of cytosolic misfolded proteins, thereby contributing to proteostasis (Gowda *et al.*, 2016).

An important question is how environmental temperature changes are registered by plants, and how monitoring such changes then leads to altered molecular responses. A recent study (Jung *et al.*, 2020) showed that a prion-like domain present in the EARLY FLOWERING 3 (ELF3) protein serves as a thermosensor in Arabidopsis. However, it is currently unclear whether temperature sensing through the ELF3 protein, or other known thermosensory mechanisms (Dai Vu *et al.*, 2019), controls nuclear splicing, and if so, which transcripts are actually affected. Addressing this will be an important aspect of future research.

Another issue that recently attracted increased interest tackles evolutionary conservation of temperature-triggered AS events in orthologous (and paralogous) genes in diverse plants (including crops), or—equally relevant—the diversification of AS in genes conserved between species. Addressing this will be essential in future research. *In silico* analysis suggests that SR proteins are evolutionarily conserved between multicellular green algae, bryophytes, and angiosperms (Melo *et al.*, 2020). In the future, experimental evidence is required to support the function of those proteins for (alternative) splicing and to investigate how they contributed to processes that allowed plants to colonize terrestrial habitats and their adaptation to terrestrial temperatures.

As described in this review, changes in environmental temperature modify the splicing patterns of many genes. A possibly important aspect that needs further investigation is how day–night changes in environmental temperature shape the AS landscape, since in their natural environment and in the field, plants are subject to frequent day–night temperature shifts. In Arabidopsis, temperature-induced AS may be involved in the regulation of the plant circadian clock (James *et al.*, 2012) and the circadian clock can also regulate AS (Yang *et al.*, 2020). Recently, an increase in daytime temperature was found to affect the splicing of the *SR45a* transcript in maize. SR45a isoforms with higher splicing efficiencies were formed later in the day (afternoon) when the temperatures rose, while earlier in the day (morning) when the temperature is lower, SR45a isoforms less efficient in splicing were produced (Li *et al.*, 2021). As plants are not able to control their ‘body’ temperature, the effects of day–night cycles on AS, besides the effect of abiotic stress-related environmental changes in temperature, may be significant. Analysing this in the future would be highly important, in particular for crops to improve their growth and yield potential at more severe day–night temperature regimes.

In order to further unravel the role of AS in response to temperature and its impact on agriculture in the context of climate change, it is important to acknowledge that even within a single species (e.g. in accessions, cultivars, mutants) there is considerable variation in the tolerance to temperature stress. In the future, detailed analyses will be required to relate differences in AS between species/accessions/cultivars to their actual temperature change response profiles.

## Conclusion

Temperature-dependent AS plays a crucial role in integrating environmental temperature information into the molecular networks that control plant biological processes, including development and growth. From a biological point of view, it seems reasonable that plants evolved AS to allow rapid adjustments of transcript abundance to changes in temperature, thereby providing developmental plasticity as seen in the regulation of flowering time by AS in response to changes in environmental temperature. Although AS is a common phenomenon in many organisms, we are only starting to understand the possibly wide relevance of AS for stress adaptation in plants. Importantly, most of the studies on temperature-induced AS have so far been conducted under controlled laboratory conditions. However, considering the huge effect climate change has on our global ecosystem and agricultural productivity it would be tremendously important to study the role of AS in natural (outdoor) settings. Only then will we be able to fully appreciate the biological role of AS in the response to changes in environmental temperature. Biotechnological approaches including, for example, CRISPR-Cas-mediated genome editing to place mutations in splice sites in order to generate the desired temperature-responsive splicing patterns, or overexpression of splice variants and siPEPs essential for improving temperature stress tolerance, could be employed to engineer crops resistant to different temperature stresses.
